# A plasma N-acetyl amino acid panel for type 2 diabetes discrimination built by targeted LC–MS/MS: A case–control study

**DOI:** 10.3389/fmed.2026.1830709

**Published:** 2026-06-03

**Authors:** Shusi Ding, Zhanxiong Xie, Daqiu Chen, Xiaomai Huang, Qiutong Wu, Lemin Zheng, Shanghua Xu, Shunxiang Luo

**Affiliations:** 1 Beijing Tiantan Hospital, China National Clinical Research Center for Neurological Diseases, Advanced Innovation Center for Human Brain Protection, Capital Medical University, Beijing, China; 2 Key Laboratory of Molecular Cardiovascular Science of Ministry of Education, NHC Key Laboratory of Cardiovascular Molecular Biology and Regulatory Peptides, Beijing Key Laboratory of Cardiovascular Receptors Research, Health Science Center, School of Basic Medical Sciences, The Institute of Cardiovascular Sciences, Peking University, Beijing, China; 3 Department of Cardiology, Nanping First Hospital Affiliated to Fujian Medical University, Nanping, Fujian, China

**Keywords:** biomarker, LC–MS/MS, metabolic signature, N-acetyl amino acids, targeted metabolomics, type 2 diabetes

## Abstract

**Background:**

Type 2 diabetes (T2D) is characterized by profound metabolic disturbances, yet clinically actionable biomarkers beyond conventional glycaemic indices remain limited. N-acetyl amino acids (NAcAAs) are emerging metabolites linked to amino acid metabolism and acetylation status, but their quantitative profile and diagnostic relevance in T2D have not been systematically defined.

**Objectives:**

To characterize the plasma NAcAA profile in T2D using a targeted LC–MS/MS approach, to evaluate associations between individual NAcAAs and clinical traits, and to develop a metabolite-based panel for T2D discrimination.

**Methods:**

In this case–control study, we developed and validated a targeted LC–MS/MS assay for the absolute quantification of 19 plasma NAcAAs. The assay was applied to 174 individuals with T2D and 68 non-diabetic controls. Global metabolic differences were assessed by principal component analysis and hierarchical clustering. Associations between NAcAAs and clinical traits were examined by correlation analysis and multivariable logistic regression. Least absolute shrinkage and selection operator (LASSO) logistic regression was used to construct a compact diagnostic signature. Model performance was evaluated by receiver operating characteristic (ROC) analysis, calibration, and decision curve analysis.

**Results:**

The plasma NAcAA landscape differed substantially between T2D and controls, with coordinated increases in metabolites such as N-acetyltryptophan and decreases in several others, including N-acetylproline, N-acetylglutamine, and N-acetyllysine. Differential NAcAAs were significantly associated with glycaemic traits, particularly fasting glucose and HbA1c, as well as renal-related markers including urea nitrogen and creatinine. In adjusted logistic regression models, N-acetyltryptophan was positively associated with T2D (OR = 9.452, 95% CI 4.421–20.211). In contrast, several NAcAAs showed inverse associations, including N-acetylproline (OR = 0.041, 95% CI 0.015–0.111) and N-acetyllysine (OR = 0.088, 95% CI 0.038–0.203). An 8-metabolite panel derived by LASSO achieved excellent discriminatory performance (AUC = 0.963, 95% CI 0.942–0.984), with good calibration and favorable net clinical benefit.

**Conclusion:**

Targeted quantitative profiling of plasma NAcAAs identifies a distinct metabolic signature associated with T2D. A compact NAcAA-based panel shows strong potential for T2D discrimination and may serve as a complementary tool for metabolic risk characterization and translational biomarker development.

## Introduction

Type 2 diabetes (T2D) continues to impose a growing global health burden, driven not only by chronic hyperglycaemia but also by heterogeneous metabolic disturbances that contribute to long-term complications (1–3). Among these complications, diabetic kidney disease is particularly common and contributes substantially to kidney failure and excess mortality (4, 5). These challenges highlight the need for biomarkers that capture early metabolic shifts and improve risk stratification beyond conventional clinical indices.

Large-scale metabolomics studies have repeatedly shown that perturbations in amino acid metabolism occur early in the course of dysglycaemia and can precede overt diabetes (6, 7). Most previous work, however, has focused on free amino acids, particularly branched-chain and aromatic amino acids. In contrast, modified amino-acid derivatives may provide additional biological information because they integrate amino acid availability with downstream processes such as acetyl-CoA metabolism, enzymatic acetylation/deacetylation, and metabolite clearance (6, 8–10).

N-acetyl amino acids (NAcAAs) are acetylated amino-acid metabolites whose circulating levels may be influenced by aminoacylase activity, acetylation status, and renal handling (11–14). Several NAcAAs have been detected in metabolomics studies related to metabolic syndrome, diabetes risk, and kidney-related phenotypes (15–18). These findings suggest that NAcAAs may represent a biologically relevant but still under-characterized class of metabolites in cardiometabolic disease. For example, plasma N-acetyltryptophan has been reported to differ in individuals with metabolic syndrome (15). In addition, multi-metabolite studies have linked amino acids and microbiota-related metabolites, including N-acetyltryptophan-related signals, to incident T2D risk (9). These observations are biologically consistent with the close interplay between tryptophan metabolism, gut microbial activity, inflammation, and insulin sensitivity (19, 20). Combined nontargeted and targeted profiling in prospective cohorts has also identified N-acetyl-L-alanine as a potential biomarker of incident diabetes (16, 17). This finding suggests that N-acetylated amino-acid metabolites can emerge as robust signals in population studies. Diet-linked metabolomics studies, including those in Mediterranean-diet settings, further suggest that amino-acid–derived metabolic patterns are modifiable and clinically informative (21). These findings support the value of quantitative profiling for translational research.

Previous metabolomics studies have mainly reported NAcAAs within broader untargeted or semi-quantitative metabolite panels. Only a limited number of NAcAA species have been examined in relation to diabetes. As a result, the plasma profile of multiple NAcAAs in T2D has not been systematically characterized using a targeted quantitative approach. In particular, it remains unclear whether a defined panel of plasma NAcAAs can capture T2D-related metabolic alterations and provide discriminatory information beyond conventional clinical indices. Therefore, targeted LC–MS/MS profiling with absolute quantification is needed to define the NAcAA landscape in T2D. This approach may improve analytical specificity, reproducibility, and translational potential (22, 23).

Against this background, we developed and applied a targeted LC–MS/MS workflow to simultaneously quantify 19 plasma N-acetyl amino acids in T2D and non-diabetic controls. By combining absolute quantification with clinical phenotyping, we pursued three aims. First, we defined the NAcAA profile associated with T2D. Second, we examined relationships between differential NAcAAs and key clinical indices, including glycaemic status, adiposity, blood pressure, lipids, and renal function. Third, we evaluated whether NAcAA panels could contribute to metabolic risk characterization in T2D.

## Methods

### Study design and participants

This hospital-based case–control study was conducted at Nanping First Hospital Affiliated to Fujian Medical University, Nanping, Fujian Province, China. Individuals with type 2 diabetes (T2D) were consecutively recruited from patients who visited or were admitted to the hospital during the study period. Non-diabetic controls were recruited from individuals undergoing routine health examination or clinical evaluation at the same hospital during the same period. Thus, both cases and controls were derived from the same source population and underwent the same fasting blood collection, plasma processing, and metabolomic detection workflow.

Eligible participants were adults aged ≥18 years with available fasting EDTA plasma samples and complete demographic, anthropometric, biochemical, and metabolomic data. T2D was defined according to a documented physician diagnosis and/or standard diagnostic laboratory criteria at enrolment. The laboratory criteria included fasting plasma glucose ≥7.0 mmol/L, HbA1c ≥ 6.5%, 2-h plasma glucose ≥11.1 mmol/L during an oral glucose tolerance test, or random plasma glucose ≥11.1 mmol/L in the presence of typical hyperglycaemic symptoms. Non-diabetic controls had no self-reported or documented history of diabetes and showed normoglycaemia at enrolment.

Participants were excluded if they had type 1 diabetes, gestational diabetes, or other specific types of diabetes. Additional exclusion criteria included diabetic ketoacidosis or hyperosmolar hyperglycaemic state at enrolment, acute infection or inflammatory disease, recent major surgery or severe trauma, malignant tumors, severe hepatic dysfunction, advanced renal dysfunction or renal replacement therapy, pregnancy or lactation, insufficient plasma volume, missing key clinical covariates, or failed metabolite quality control. After applying these criteria, 174 individuals with T2D and 68 non-diabetic controls were included in the final analysis.

The study protocol was approved by the Medical Ethics Committee of Nanping First Hospital (approval no. NPSY202503007), and all participants provided written informed consent.

### Clinical assessment and biochemical measurements

Demographic and anthropometric information was collected at enrolment. Body mass index (BMI) was calculated as weight divided by height squared (kg/m^2^). Blood pressure was measured in the seated position after at least 5 min of rest, and systolic blood pressure (SBP) and diastolic blood pressure (DBP) were recorded. After an overnight fast, venous blood was collected for routine laboratory testing using standard clinical laboratory methods. The measured variables included fasting glucose, glycated haemoglobin (HbA1c), triacylglycerol (TG), total cholesterol, HDL cholesterol, LDL cholesterol, urea nitrogen, and creatinine.

For participants with T2D, information on diabetes duration and antidiabetic medication use was extracted from medical records when available. Diabetes duration was categorized as newly diagnosed, <1 year, 1 to <5 years, 5 to <10 years, and ≥10 years. Antidiabetic medication use was summarized from regular or current medication records. Medication categories included metformin, insulin, alpha-glucosidase inhibitors, SGLT2 inhibitors, DPP-4 inhibitors, sulfonylurea/glinides, and GLP-1 receptor agonists. Detailed information is provided in Table 1. Detailed dietary intake was not systematically recorded in this cohort; however, all blood samples for biochemical and metabolomic analyses were collected after an overnight fast to minimize acute postprandial dietary effects.

**Table 1:** Diabetes duration and antidiabetic medication use among T2D participants.

Domain	Variable	Value
Diabetes duration	Duration information available	174
	Newly diagnosed	13 (7.5%)
	<1 year	13 (7.5%)
	1 to <5 years	30 (17.2%)
	5 to <10 years	31 (17.8%)
	≥10 years	87 (50.0%)
Antidiabetic treatment	Any antidiabetic treatment recorded	111 (63.8%)
	No antidiabetic treatment recorded	60 (34.5%)
	Treatment status not clearly recorded	3 (1.7%)
Medication categories	Medication information available from regular/current medication records	111
	Metformin	66 (37.9%)
	Insulin	55 (31.6%)
	Alpha-glucosidase inhibitor	55 (31.6%)
	SGLT2 inhibitor	32 (18.4%)
	DPP-4 inhibitor	31 (17.8%)
	Sulfonylurea/glinide	32 (18.4%)
	GLP-1 receptor agonist	9 (5.2%)

T2D, type 2 diabetes; IQR, interquartile range; SD, standard deviation; SGLT2, sodium-glucose cotransporter 2; DPP-4, dipeptidyl peptidase-4; GLP-1, glucagon-like peptide-1. Percentages were calculated using the total number of T2D participants (*n* = 174) as the denominator unless otherwise specified. Medication categories were not mutually exclusive because some participants received combination therapy.

### Plasma collection and handling

Fasting blood for metabolomics was collected into EDTA anticoagulant tubes. Plasma was separated by refrigerated centrifugation, aliquoted to minimize freeze–thaw cycles, and stored at −80 °C until analysis. Samples from T2D and control participants were randomized across analytical runs. A pooled quality-control (QC) sample was prepared by combining small aliquots of study plasma and was injected periodically to monitor analytical stability.

### Targeted LC–MS/MS quantification of N-acetyl amino acids

For sample preparation, plasma aliquots were thawed on ice and gently mixed. An aliquot of plasma (1 volume) was precipitated with 4 volumes of pre-chilled methanol (−20 °C) to achieve a 1:4 (plasma:methanol, v/v) ratio. The methanol used for protein precipitation contained a mixed internal-standard solution. The solution consisted of isotope-labelled amino acids corresponding to the amino-acid backbones of the target NAcAAs. The internal-standard panel included Glycine-D2, L-Phenylalanine-D8, L-Tryptophan-D8, L-Leucine-D10, L-Methionine-D8, L-Proline-D7, L-Tyrosine-D7, L-Valine-D8, L-Cysteine-D3, L-Alanine-D4, L-Threonine-D5, L-Glutamic acid-D5, L-Aspartic acid-D3, L-Glutamine-D5, L-Serine-D3, L-Asparagine-D3, L-Histidine-D5, L-Lysine-D8, and L-Arginine-D7. Internal-standard correction was applied to reduce variation related to extraction efficiency and instrumental response. The retention times and MRM parameters of all internal standards are summarized in Table 2, and representative extracted-ion chromatograms are shown in Supplementary Figure S1. The mixture was vortexed thoroughly and incubated on ice to facilitate protein precipitation. Samples were then centrifuged at 16,000 × g for 20 min at 4 °C, and the clear supernatant was carefully transferred to autosampler vials without disturbing the pellet. The resulting extracts were analysed by LC–MS/MS in MRM mode. Compound-specific MRM transitions and optimized MS parameters (including precursor/product ion pairs, declustering potentials, and collision energies) are summarized in Table 3.

**Table 2:** Isotope-labelled internal standards used for LC–MS/MS quantification.

ID	RT (min)	Q1 Mass (Da)	Q3 Mass (Da)	DP (volts)	CE (volts)
L-Phenylalanine-D8	2.43	174.1	128.1	90	24
L-Tryptophan-D8	2.61	213.1	153.1	95	26
L-Leucine-D10	3.66	142.1	96.1	85	24
L-Methionine-D8	3.98	158.1	112.1	90	26
L-Proline-D7	4.73	123.1	77.1	70	22
L-Tyrosine-D7	4.82	189.1	143.1	90	26
L-Valine-D8	4.95	126.1	80.1	70	24
L-Cysteine-D3	11.14	125.0	79.1	80	24
L-Alanine-D4	7.04	94.1	48.1	70	22
L-Threonine-D5	7.12	125.1	79.1	75	24
L-Glutamic acid-D5	7.22	153.1	89.1	80	24
L-Aspartic acid-D3	7.31	137.0	77.1	80	24
Glycine-D2	7.44	78.0	78.0	70	20
L-Glutamine-D5	7.61	152.1	89.1	85	26
L-Serine-D3	7.52	109.0	63.1	80	22
L-Asparagine-D3	8.22	136.1	77.1	85	24
L-Histidine-D5	12.22	161.1	115.1	90	24
L-Lysine-D8	12.94	155.1	92.1	90	24
L-Arginine-D7	14.21	182.1	77.1	90	24

Retention time (RT), precursor/product ion transitions, declustering potential (DP), and collision energy (CE) are shown for each isotope-labelled internal standard used in the targeted LC–MS/MS assay.

**Table 3:** MRM transitions and optimized MS parameters for 19 NAcAAs.

ID	Retention time (min)	Q1 Mass (Da)	Q3 Mass (Da)	DP (volts)	CE (volts)
Acetyl-leucine	2.04	174.1	132.1	60	12
Acetyl-valine	2.21	160.1	118.1	55	12
Acetyl-glycine	7.25	118.1	76	50	16
Acetyl-methionine	2.62	192.1	150.1	60	12
Acetyl-glutamic acid	12.72	190.2	147.2	29	19
Acetyl-tryptophan	3.28	247.2	159.1	35	25
Acetyl-alanine	3.13	132.1	44.1	45	14
Acetyl-asparagine	8.10	175.1	87.1	55	18
Acetyl-aspartic acid	8.28	176.1	134	50	12
Acetyl-glutamine	12.41	189.1	101.1	55	18
Acetyl-histidine	11.32	198.2	110.1	32	22
Acetyl-serine	10.01	148.1	60	50	16
Acetyl-proline	4.31	158.1	70.1	55	18
Acetyl-threonine	7.47	162.1	74	50	16
Acetyl-cysteine	3.65	164.1	122	55	12
Acetyl-lysine	5.15	189.2	84.1	31	21
Acetyl-arginine	14.23	217.2	116.1	34	24
Acetyl-tyrosine	7.07	224.2	136.1	33	23
Acetyl-phenylalanine	1.86	208.1	120.3	60	25

Precursor/product ion pairs (Q1/Q3), declustering potential (DP), and collision energy (CE) optimized for each analyte under the LC–MS/MS MRM method.

We quantified 19 N-acetyl amino acids (NAcAAs) in plasma on a SCIEX 4500 triple-quadrupole mass spectrometer operating in multiple reaction monitoring (MRM) mode. The mass spectrometer was equipped with a Turbo Spray electrospray ionization source and operated in positive-ion mode. The optimized source parameters were set as follows: ion spray voltage, 5,500 V; source temperature, 600 °C; curtain gas, 35 psi; collision gas, 7; ion source gas 1, 60 psi; and ion source gas 2, 60 psi. Entrance potential and collision cell exit potential were set at 10 V and 7 V, respectively. Compound-specific MRM transitions and optimized parameters, including precursor/product ion pairs, declustering potential, and collision energy, are summarized in Table 3. Chromatographic separation was performed on an Intrada Amino Acid column (150 mm × 3.0 mm, 3 μm). Mobile phase A consisted of acetonitrile containing 0.1% formic acid, and mobile phase B consisted of acetonitrile/100 mM ammonium formate (20/80, v/v). A stepwise gradient was applied over a 16-min run: 5% B (0–1.0 min; 0.6 mL/min), 20% B at 8.0 min, 70% B at 12.0 min, and 100% B at 14.0 min, followed by a hold to 16 min. The system was returned to initial conditions before the next injection.

### Calibration and method validation

Quantification was performed using external calibration curves prepared from authentic standards spanning the concentration ranges used for each analyte (Table 4). Calibration standards, study samples, and QC samples were processed in parallel using the same preparation procedure, and internal-standard correction was applied to control for extraction and instrument variability. Calibration curves were fitted using weighted linear regression, and concentrations were reported in μM.

**Table 4:** Analytical performance of the targeted LC–MS/MS assay for plasma NAcAAs.

ID	Accuracy low QC (%)	Accuracy high QC (%)	Carry-over (%)	LOD (μM)	LOQ (μM)	Linearity (μM)	r^2^	Repeatability (CV%)	Reproducibility (CV%)
Acetyl-phenylalanine	98.9	96.5	0.03	0.00027	0.00082	0.005–50	0.9965	4.8	4.2
Acetyl-leucine	98.7	102.8	0.05	0.03	0.091	0.5–50	0.9946	1.5	8.5
Acetyl-valine	106.3	93.7	0.11	0.00052	0.0016	0.05–50	0.9832	13	13.2
Acetyl-glycine	90.4	109.4	0.02	0.0033	0.01	0.1–50	0.9871	4.5	12.6
Acetyl-methionine	116.8	88.6	0.04	0.00011	0.00033	0.005–10	0.9867	4.4	4
Acetyl-glutamic acid	95.2	115.3	0.34	0.0037	0.0109	0.1–50	0.9779	1.5	15.7
Acetyl-tryptophan	103.1	99.2	0.28	0.002	0.006	0.01–50	0.9865	1.1	3.7
Acetyl-alanine	95.6	105.7	0.07	0.0042	0.0126	0.2–50	0.9841	4.3	22.8
Acetyl-asparagine	97.4	91.4	0.13	0.00027	0.00081	0.02–10	0.9882	11	11.4
Acetyl-aspartic acid	110.2	111.6	0.03	0.0043	0.013	0.2–50	0.9842	3	8.9
Acetyl-glutamine	90.1	97.8	0.16	0.00094	0.0028	0.05–50	0.9779	6.7	13.1
Acetyl-histidine	93.5	100.5	0.15	0.00016	0.00048	0.02–10	0.9917	3.7	31.5
Acetyl-serine	101.9	89.3	0.06	0.0056	0.0166	0.05–50	0.9919	9.6	4.1
Acetyl-proline	113.5	118.2	0.09	0.0156	0.0468	0.1–50	0.9869	12.5	23.5
Acetyl-threonine	94.8	94.6	0.22	0.0104	0.0313	0.2–50	0.9887	3.7	3.6
Acetyl-cysteine	94.2	107.1	0.14	0.0345	0.1034	0.02–50	0.9875	4.6	7.9
Acetyl-lysine	108.9	92.9	0.03	0.0726	0.217	0.1–50	0.9912	4.9	11.8
Acetyl-arginine	91.7	104.3	0.08	0.00016	0.00047	0.01–10	0.9889	9.1	8.2
Acetyl-tyrosine	120.6	112.7	0.1	0.00012	0.00037	0.05–50	0.9901	11.5	9.8

Assay accuracy, carry-over, limits of detection (LOD) and quantification (LOQ), calibration linearity range and correlation coefficient (r^2^), and precision metrics (repeatability and reproducibility, expressed as CV%) for each NAcAA.

Method performance was assessed using commonly applied bioanalytical validation criteria, including accuracy at low and high QC levels, carry-over, linearity, lower limit of detection (LOD), lower limit of quantification (LOQ), repeatability, and reproducibility. Within-run and between-run precision was evaluated using QC samples at multiple concentration levels. Key performance characteristics are summarized in Table 4. Matrix effects were evaluated at low and high QC levels by comparing analyte responses in post-extraction spiked plasma matrix with those in neat standard solutions at the same concentrations. Internal-standard-normalized matrix effects were further calculated using the corresponding isotope-labelled internal standards to assess whether matrix-related ion suppression or enhancement was effectively corrected. The results of plasma matrix-effect evaluation are summarized in Table 5.

**Table 5:** Evaluation of plasma matrix effects for 19 N-acetyl amino acids at low and high quality-control levels.

ID	Low QC (μM)	ME at low QC, mean ± SD (%)	IS-normalized ME at low QC, mean ± SD (%)	CV at low QC (%)	High QC (μM)	ME at high QC, mean ± SD (%)	IS-normalized ME at high QC, mean ± SD (%)	CV at high QC (%)
Acetyl-phenylalanine	0.02	91.8 ± 5.0	99.2 ± 4.1	4.1	40	94.5 ± 3.8	100.5 ± 3.0	3.0
Acetyl-leucine	1	95.6 ± 4.7	101.0 ± 3.6	3.6	40	98.2 ± 3.2	100.8 ± 2.5	2.5
Acetyl-valine	0.2	93.4 ± 5.1	99.6 ± 4.2	4.2	40	96.9 ± 3.5	100.2 ± 2.8	2.8
Acetyl-glycine	0.5	88.7 ± 6.0	98.5 ± 4.9	5.0	40	91.5 ± 4.4	99.3 ± 3.6	3.6
Acetyl-methionine	0.02	90.5 ± 5.5	99.0 ± 4.6	4.6	8	93.7 ± 4.1	100.1 ± 3.3	3.3
Acetyl-glutamic acid	0.5	84.6 ± 6.6	97.4 ± 5.7	5.9	40	88.8 ± 5.0	98.8 ± 4.2	4.3
Acetyl-tryptophan	0.05	89.2 ± 5.3	98.7 ± 4.4	4.5	40	92.4 ± 3.9	99.7 ± 3.2	3.2
Acetyl-alanine	0.5	92.1 ± 5.2	99.8 ± 4.3	4.3	40	95.0 ± 3.6	100.4 ± 2.9	2.9
Acetyl-asparagine	0.1	86.2 ± 6.4	98.1 ± 5.4	5.5	8	89.5 ± 4.9	99.0 ± 4.0	4.0
Acetyl-aspartic acid	0.5	83.8 ± 6.8	97.2 ± 5.9	6.1	40	87.6 ± 5.3	98.5 ± 4.4	4.5
Acetyl-glutamine	0.2	87.5 ± 6.1	98.0 ± 5.1	5.2	40	91.0 ± 4.6	99.1 ± 3.8	3.8
Acetyl-histidine	0.1	85.9 ± 6.9	97.8 ± 6.0	6.1	8	89.2 ± 5.1	99.2 ± 4.1	4.1
Acetyl-serine	0.2	88.4 ± 6.2	98.4 ± 5.2	5.3	40	91.8 ± 4.5	99.4 ± 3.7	3.7
Acetyl-proline	0.5	94.7 ± 4.8	100.7 ± 3.9	3.9	40	97.8 ± 3.3	100.9 ± 2.7	2.7
Acetyl-threonine	0.5	89.5 ± 5.8	98.9 ± 4.8	4.9	40	93.0 ± 4.2	99.8 ± 3.4	3.4
Acetyl-cysteine	0.5	82.9 ± 7.1	96.8 ± 6.4	6.6	40	86.5 ± 5.5	98.2 ± 4.6	4.7
Acetyl-lysine	1	86.8 ± 6.6	98.3 ± 5.5	5.6	40	90.1 ± 5.0	99.5 ± 4.0	4.0
Acetyl-arginine	0.05	84.1 ± 7.2	97.6 ± 6.2	6.4	8	88.0 ± 5.4	98.7 ± 4.5	4.6
Acetyl-tyrosine	0.2	90.7 ± 5.4	99.1 ± 4.5	4.5	40	93.9 ± 4.0	100.0 ± 3.2	3.2

QC, quality control; ME, matrix effect; IS, internal standard; CV, coefficient of variation; SD, standard deviation. Low QC and high QC indicate the spiked concentration levels used for matrix-effect evaluation. ME (%) was calculated by comparing the analyte response in post-extraction spiked plasma matrix with that in neat standard solution at the same concentration. IS-normalized ME (%) was calculated after normalization to the corresponding isotope-labeled internal standard. Data are presented as mean ± SD (%), and CV (%) represents the variability of the IS-normalized matrix effect.

### Statistical analysis and model development

Continuous variables are presented as mean ± standard deviation (SD) or median (interquartile range) according to distribution, and categorical variables as counts and percentages. Between-group comparisons were performed using Student’s t-test or the Mann–Whitney U test for continuous variables and the chi-square test for categorical variables.

To describe global NAcAA patterns, metabolite concentrations were standardized to z-scores (and log-transformed when needed for symmetry) before principal component analysis (PCA) and heatmap visualization. Heatmaps were generated using z-scored metabolites with hierarchical clustering of samples and metabolites. Correlations between differential NAcAAs and clinical indices were evaluated using Spearman correlation coefficients.

Associations between individual NAcAAs and T2D were examined using logistic regression, with odds ratios (ORs) and 95% confidence intervals (CIs) reported. Model 1 was adjusted for age, sex, and BMI; Model 2 was additionally adjusted for SBP, TG, HDL cholesterol, and creatinine. To construct a compact diagnostic signature, we applied least absolute shrinkage and selection operator (LASSO) logistic regression to the full set of 19 NAcAAs. Ten-fold cross-validation was used to select the optimal penalty parameter (*λ*). The selected metabolites were then entered into a multivariable logistic regression model to generate predicted probabilities.

Model discrimination was evaluated using receiver operating characteristic (ROC) curves and the area under the curve (AUC) with 95% confidence intervals. Calibration was assessed by comparing predicted and observed probabilities, with bootstrap resampling used for internal correction. Decision curve analysis (DCA) was performed to evaluate the net clinical benefit of models across a range of risk thresholds. All tests were two-sided, and *p* < 0.05 was considered statistically significant.

## Results

### Targeted LC–MS/MS profiling of 19 N-acetyl amino acids

**Figure 1:**
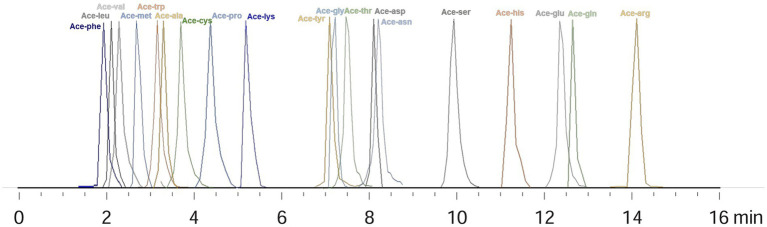
Targeted LC–MS/MS profiling of 19 *N*-acetyl amino acids (NAcAAs). Representative extracted-ion chromatograms acquired in multiple reaction monitoring (MRM) mode on the SCIEX 4500 platform, showing baseline-resolved peaks and stable retention for the 19 targeted NAcAAs within a 16 min run. Peaks are annotated with compound names.

We developed an integrated LC–MS/MS workflow to simultaneously quantify 19 N-acetyl amino acids (NAcAAs) in plasma on a SCIEX 4500 triple-quadrupole platform operating in multiple reaction monitoring (MRM) mode. To maximize retention and peak shape for these polar NAcAAs, we employed a high-organic starting condition and optimized a volatile buffer system compatible with electrospray ionization. Chromatographic separation was achieved on an Intrada Amino Acid column (150 mm × 3.0 mm, 3 μm). Mobile phase A consisted of acetonitrile containing 0.1% formic acid, and mobile phase B consisted of acetonitrile/100 mM ammonium formate (20/80, v/v). A stepwise gradient was applied over a 16 min run, starting at 5% B (0–1.0 min; 0.6 mL/min), increasing to 20% B at 8.0 min, 70% B at 12.0 min, and finally to 100% B at 14.0 min with a hold to 16 min. Under these conditions, representative extracted-ion chromatograms showed sharp and well-resolved peaks with stable retention for the target NAcAAs (Figure 1). Compound-specific MRM transitions and optimized MS parameters (including precursor/product ion pairs, declustering potentials, and collision energies) are summarized in Table 3. In addition to the analyte MRM transitions, the full internal-standard information, including retention time, Q1/Q3 transitions, DP, and CE, is provided in Table 2. Representative extracted-ion chromatograms of the internal-standard panel are shown in Supplementary Figure S1. The validation performance metrics further support the robustness of the quantitative method, as summarized in Table 4. In addition, the plasma matrix-effect evaluation showed that the internal-standard-normalized matrix effects were close to 100% at both low and high QC levels, supporting the suitability of the assay for plasma sample analysis (Table 5).

### Baseline characteristics

Baseline characteristics of the study participants are summarized in Table 6, including 68 healthy controls and 174 individuals with T2D. The sex distribution was comparable between groups (male: 34/68 vs. 93/174, *p* = 0.629). Age was also comparable between the control and T2D groups (60.5 [52, 71] vs. 62 [54, 72] years, *p* = 0.743), whereas BMI was slightly higher in participants with T2D (24.203 ± 3.5675 vs. 23.363 ± 0.93696 kg/m^2^, *p* = 0.005). Both systolic and diastolic blood pressure were higher in the T2D group (SBP: 132.5 [122, 148.75] vs. 119.5 [118, 123] mmHg; DBP: 82 [74, 88] vs. 77 [76, 79] mmHg; both *p* < 0.001). As expected, glycaemic indices were markedly elevated in T2D, including fasting glucose (8.455 [6.375, 12.35] vs. 5.09 [4.8025, 5.375] mmol/L, *p* < 0.001) and HbA1c (9.45 [7.5, 10.875] vs. 5.7 [5.575, 5.9] %, *p* < 0.001). For lipid profiles, triacylglycerol, total cholesterol, and LDL did not differ significantly between groups, whereas HDL was lower in T2D (1.135 [0.94, 1.335] vs. 1.255 [1.0475, 1.48] mmol/L, p < 0.001). Regarding renal-related markers, T2D participants showed higher urea nitrogen (6.79 [5.3525, 8.76] vs. 4.765 [4.2325, 5.45] mmol/L, *p* < 0.001) and creatinine (87.25 [68.225, 104.42] vs. 76.3 [63.75, 86.05] μmol/L, *p* < 0.001).

**Table 6:** Baseline characteristics of healthy controls and participants with T2D.

Characteristics	Healthy controls	T2D	*p* value
*n*	68	174	
Sex, *n* (%)			0.629
Male	34 (50%)	93 (53.4%)	
Female	34 (50%)	81 (46.6%)	
Age (years)	60.5 (52, 71)	62 (54, 72)	0.743
BMI (kg/m^2^)	23.363 ± 0.93696	24.203 ± 3.5675	0.005
Systolic (mmHg)	119.5 (118, 123)	132.5 (122, 148.75)	< 0.001
Diastolic (mmHg)	77 (76, 79)	82 (74, 88)	< 0.001
Fasting glucose (mmol/L)	5.09 (4.8025, 5.375)	8.455 (6.375, 12.35)	< 0.001
HbA1c, %	5.7 (5.575, 5.9)	9.45 (7.5, 10.875)	< 0.001
Triacylglycerol (mmol/L)	1.64 (1.2125, 2.4575)	1.515 (1.17, 2.33)	0.607
Total cholesterol (mmol/L)	5.0616 ± 1.1162	4.8359 ± 1.4218	0.194
HDL (mmol/L)	1.255 (1.0475, 1.48)	1.135 (0.94, 1.335)	< 0.001
LDL (mmol/L)	2.995 (2.565, 3.74)	2.85 (2.0725, 3.6125)	0.181
Urea nitrogen (mmol/L)	4.765 (4.2325, 5.45)	6.79 (5.3525, 8.76)	< 0.001
Creatinine (μmol/L)	76.3 (63.75, 86.05)	87.25 (68.225, 104.42)	< 0.001

Diabetes duration and antidiabetic medication use among T2D participants are summarized in Table 1. Diabetes duration information was available for all 174 T2D participants. Thirteen participants were newly diagnosed, 13 had diabetes duration of <1 year, 30 had 1 to <5 years, 31 had 5 to <10 years, and 87 had ≥10 years of diabetes. Antidiabetic treatment was recorded in 111 participants, whereas 60 had no antidiabetic treatment recorded and 3 had unclear treatment status. The most frequently recorded medication categories were metformin, insulin, alpha-glucosidase inhibitors, SGLT2 inhibitors, DPP-4 inhibitors, sulfonylurea/glinides, and GLP-1 receptor agonists. Because several participants received combination therapy, medication categories were not mutually exclusive.

### Global plasma NAcAA profile in T2D and controls

**Figure 2:**
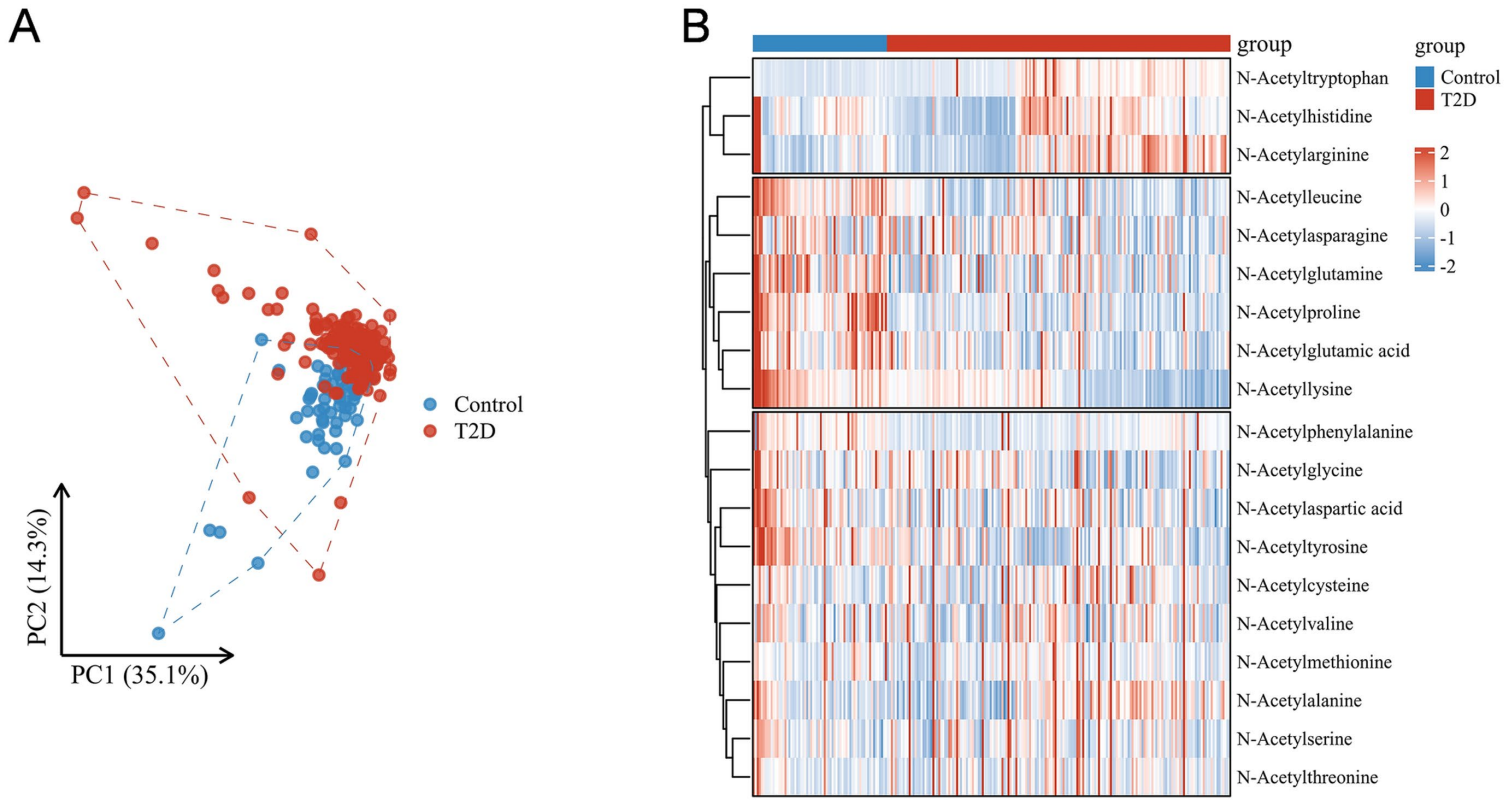
Global separation and clustering of plasma NAcAA profiles between healthy controls and T2D. **(A)** Principal component analysis (PCA) score plot based on the quantified plasma NAcAA concentrations. Each point represents one participant, with controls shown in blue and individuals with T2D shown in red. PC1 and PC2 explain 35.1 and 14.3% of the total variance in the NAcAA dataset, respectively, together accounting for 49.4% of the overall metabolic variation. The dashed polygons indicate the distribution range of each group. T2D samples showed a partial shift from controls, mainly along PC1, suggesting that the overall plasma NAcAA profile differs between groups. **(B)** Heatmap showing standardized NAcAA abundance across participants. Rows represent individual NAcAAs and columns represent participants. Metabolite concentrations were standardized as *z*-scores before visualization; red indicates higher relative abundance and blue indicates lower relative abundance compared with the mean level of each metabolite. The top annotation bar indicates group membership, with controls in blue and T2D in red. Unsupervised hierarchical clustering of metabolites revealed distinct NAcAA clusters with coordinated changes.

Targeted LC–MS/MS quantified 19 N-acetyl amino acids (NAcAAs) in plasma from the control and T2D groups. Principal component analysis (PCA) based on the 19 NAcAAs showed partial separation between groups, with the first two components explaining 35.1% (PC1) and 14.3% (PC2) of the total variance (Figure 2A). Although an overlap remained, T2D samples tended to shift along PC1 relative to controls, suggesting an overall difference in the NAcAA pattern. Consistently, unsupervised hierarchical clustering of z-scored NAcAA concentrations revealed coordinated metabolite changes across individuals (Figure 2B). One metabolite cluster, including N-acetyltryptophan, N-acetylhistidine, and N-acetylarginine, showed higher standardized abundance in T2D. Another cluster, including N-acetylleucine, N-acetylasparagine, N-acetylglutamine, N-acetylproline, N-acetylglutamic acid, and N-acetyllysine, tended to show lower abundance in T2D. These patterns suggest structured remodeling of the NAcAA landscape rather than isolated single-metabolite shifts.

**Figure 3:**
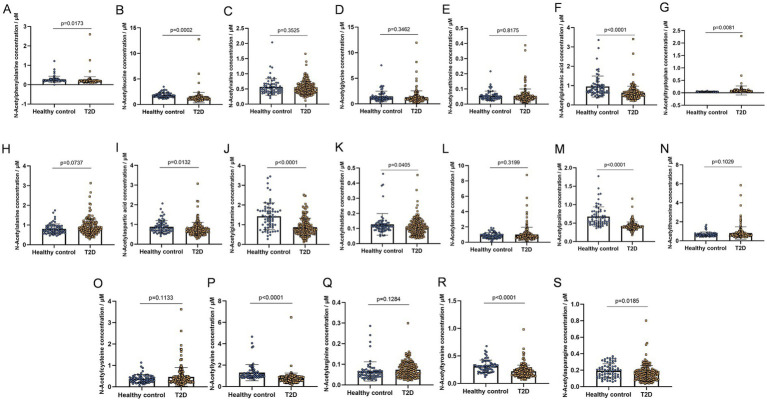
Group-wise comparison of plasma NAcAA concentrations. Scatter/box plots of plasma concentrations for the 19 NAcAAs (**A–S**, as labeled in the figure) comparing healthy controls and T2D. Each dot represents one participant; boxes indicate the interquartile range with the median line. Data were analyzed by unpaired Student’s *t*-test.

We next examined each NAcAA individually (Figure 3). Overall, multiple NAcAAs displayed between-group shifts with inter-individual variability. Consistent with the clustering results, N-acetyltryptophan, N-acetylhistidine, and N-acetylarginine were higher in T2D. In contrast, N-acetylleucine, N-acetylasparagine, N-acetylglutamine, N-acetylproline, N-acetylglutamic acid, and N-acetyllysine tended to be lower in T2D than in controls. The remaining NAcAAs (N-acetylphenylalanine, N-acetylglycine, N-acetylaspartic acid, N-acetyltyrosine, N-acetylcysteine, N-acetylvaline, N-acetylmethionine, N-acetylalanine, N-acetylserine, and N-acetylthreonine) showed smaller shifts with substantial overlap between groups, suggesting that the group difference is driven by coordinated changes across a subset of the NAcAA panel rather than uniform alteration across all metabolites.

### Clinical relevance and diagnostic performance of the NAcAA-based model

**Figure 4:**
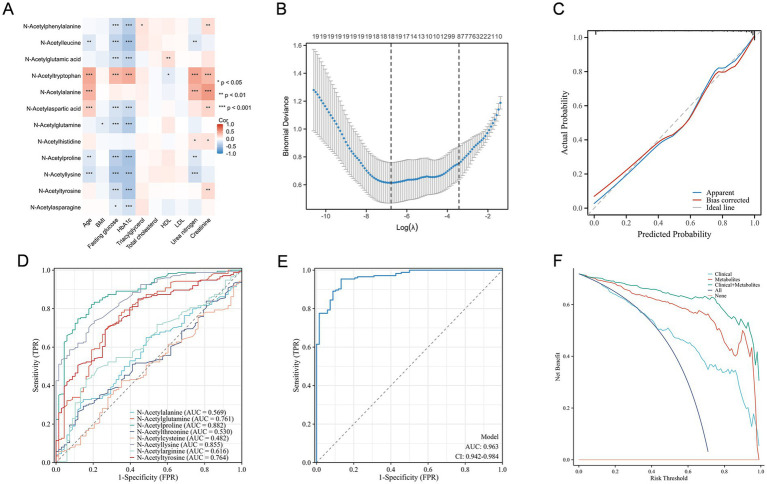
Correlation structure and model development for T2D discrimination. **(A)** Correlation heatmap between differential NAcAAs and clinical indices (age, BMI, fasting glucose, HbA1c, triacylglycerol, total cholesterol, HDL, LDL, urea nitrogen, and creatinine). Colors denote correlation coefficients, and significance is indicated by *p < 0.05, **p < 0.01, and ***p < 0.001. **(B)** Ten-fold cross-validated binomial deviance curve for LASSO logistic regression; dashed vertical lines indicate the selected tuning parameters (λ_min and λ_1se). **(C)** Calibration plot of the combined model showing apparent and bias-corrected calibration against the ideal reference line. **(D)** Receiver operating characteristic (ROC) curves for the eight candidate NAcAAs (*N*-acetylalanine, *N*-acetylglutamine, *N*-acetylproline, *N*-acetylthreonine, *N*-acetylcysteine, *N*-acetyllysine, *N*-acetylarginine, and *N*-acetyltyrosine); AUCs are shown in the legend. **(E)** ROC curve for the final combined model derived from the selected NAcAA panel; AUC and 95% CI are reported in the panel. **(F)** Decision curve analysis (DCA) comparing net benefit across risk thresholds for the clinical model, metabolite model, and combined model, with “treat-all” and “treat-none” strategies as references.

Correlation analysis showed that the targeted NAcAAs were not uniformly related to clinical traits (Figure 4A). Fasting glucose and HbA1c displayed significant inverse correlations with several NAcAAs, including N-acetylphenylalanine, N-acetylleucine, N-acetylglutamic acid, N-acetylaspartic acid, N-acetylglutamine, N-acetylproline, N-acetyllysine, N-acetyltyrosine and N-acetylasparagine. In contrast, N-acetyltryptophan showed a clear positive correlation with both fasting glucose and HbA1c. Renal-related indices also showed structured associations. Urea nitrogen and creatinine were positively correlated with N-acetyltryptophan and N-acetylalanine. Conversely, urea nitrogen was inversely correlated with N-acetylleucine, N-acetylproline and N-acetyllysine. Lipid traits showed fewer and generally weaker links.

To build a compact diagnostic signature, we applied LASSO logistic regression to the NAcAA features (Figure 4B). Eight metabolites were retained for the metabolite panel (N-acetylalanine, N-acetylglutamine, N-acetylproline, N-acetylthreonine, N-acetylcysteine, N-acetyllysine, N-acetylarginine, and N-acetyltyrosine). In single-marker ROC analyses, the best discrimination was observed for N-acetylproline (AUC = 0.882) and N-acetyllysine (AUC = 0.855), followed by N-acetyltyrosine (AUC = 0.764) and N-acetylglutamine (AUC = 0.761); the remaining metabolites showed modest performance (AUC range 0.482–0.616) (Figure 4D). When combined into the multivariable NAcAA panel model, discrimination improved substantially (AUC = 0.963, 95% CI 0.942–0.984; Figure 4E). For clinical contextualization, a conventional glycaemic-marker model combining fasting plasma glucose and HbA1c achieved an AUC of 0.908, which was lower than that of the NAcAA-based metabolite panel (Supplementary Figure S2A). The calibration plot showed close agreement between predicted and observed probabilities after bias correction, suggesting stable model calibration (Figure 4C). In decision curve analysis, the clinical + metabolites model provided a higher net benefit than the clinical-only or metabolites-only model across a wide range of threshold probabilities (Figure 4F), supporting potential clinical usefulness.

### Associations of individual N-acetyl amino acids with T2D

**Figure 5:**
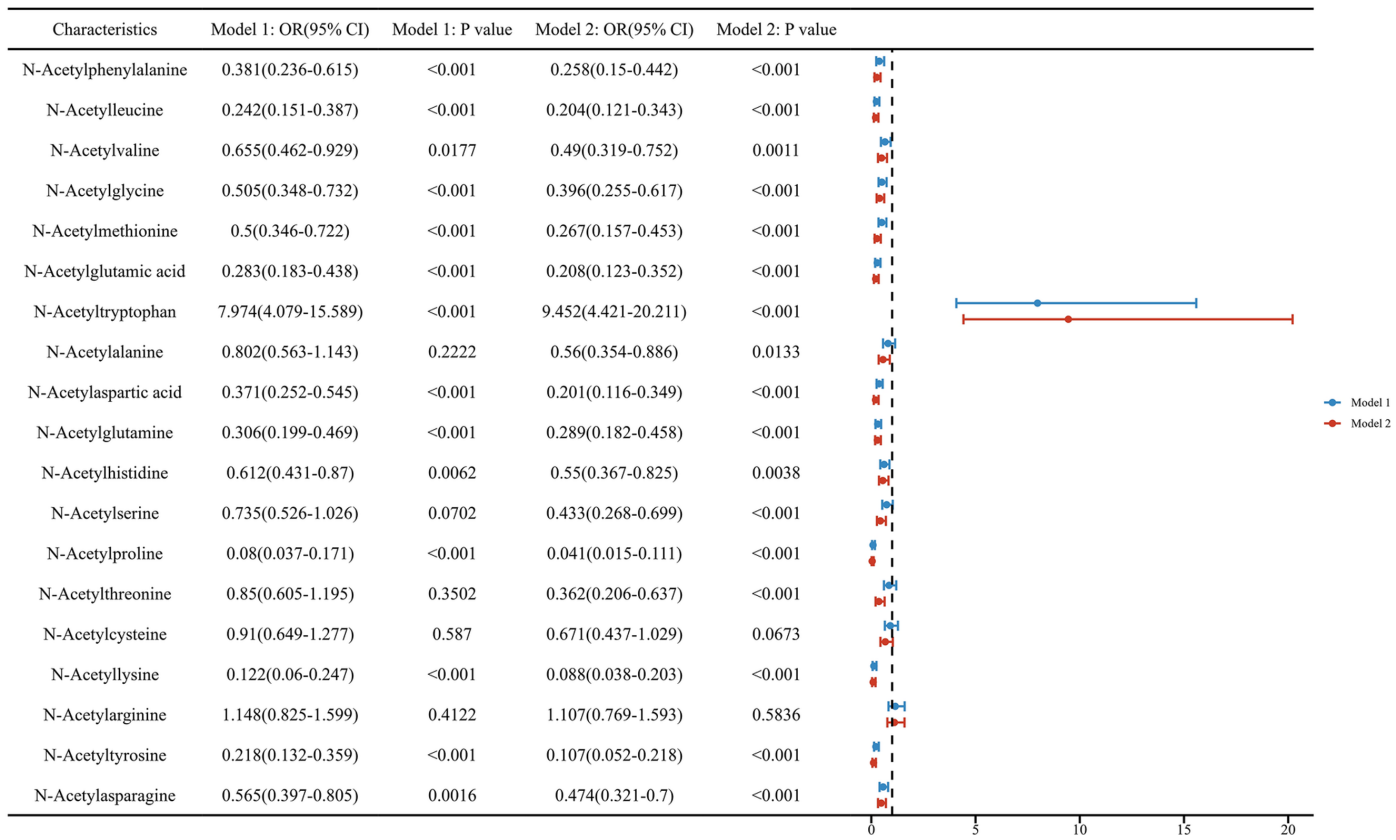
Associations between individual NAcAAs and odds of T2D. Odds ratios (ORs) with 95% confidence intervals from logistic regression models are shown for each NAcAA. Model 1 is adjusted for age, sex, and BMI. Model 2 is additionally adjusted for systolic blood pressure (SBP), triacylglycerol (TG), HDL, and creatinine.

As shown in Figure 5A, most N-acetyl amino acids (NAcAAs) were inversely associated with T2D in both models. In Model 1, 14 of 19 NAcAAs showed significant associations with lower odds of T2D. The strongest inverse association was observed for N-acetylproline (OR 0.080, 95% CI 0.037–0.171; *p* < 0.001), followed by N-acetyllysine (OR 0.122, 95% CI 0.060–0.247; p < 0.001) and N-acetyltyrosine (OR 0.218, 95% CI 0.132–0.359; p < 0.001). In contrast, N-acetyltryptophan was positively associated with T2D (OR 7.974, 95% CI 4.079–15.589; p < 0.001), indicating a markedly higher odds of T2D with increasing N-acetyltryptophan levels.

After applying Model 2, the overall pattern and direction of associations remained consistent. Seventeen NAcAAs were significantly associated with T2D, and several metabolites that were not significant in Model 1 became significant in Model 2, including N-acetylalanine (OR 0.560, 95% CI 0.354–0.886; *p* = 0.013), N-acetylserine (OR 0.433, 95% CI 0.268–0.699; *p* < 0.001), and N-acetylthreonine (OR 0.362, 95% CI 0.206–0.637; p < 0.001). N-acetylcysteine and N-acetylarginine also remained non-significant. Notably, the positive association for N-acetyltryptophan persisted and appeared even stronger in Model 2 (OR 9.452, 95% CI 4.421–20.211; *p* < 0.001).

## Discussion

In this targeted metabolomics study, we established an LC–MS/MS workflow for absolute quantification of 19 N-acetyl amino acids (NAcAAs) and applied it to plasma from 68 healthy controls and 174 individuals with type 2 diabetes (T2D). The global NAcAA profile clearly differed between groups, and several NAcAAs showed consistent shifts and clinically relevant correlations with glycaemic and renal traits. In multivariable analyses, most NAcAAs were inversely associated with T2D, whereas N-acetyltryptophan showed a strong positive association. A compact eight-metabolite signature derived by LASSO provided excellent discrimination (AUC 0.963, 95% CI 0.942–0.984). The model also showed close agreement between predicted and observed risks and provided favourable net benefit across a wide range of threshold probabilities. Several metabolites showed strong associations with T2D in logistic regression analysis. All metabolite concentrations were log-transformed and Z-score standardized before modeling. Therefore, the odds ratios represent the effect per one standard deviation increase in the transformed metabolite level, rather than the effect of a one-unit increase in the original concentration. In addition, each metabolite was modeled separately rather than being simultaneously included with all detected metabolites, which reduced the risk of overfitting caused by excessive predictors and multicollinearity. Although this modeling strategy reduced the risk of overfitting, the possibility of model instability due to the relatively limited sample size cannot be completely excluded. The strong inverse association between N-acetylproline and T2D may reflect a pronounced alteration in amino acid-related metabolic remodeling in T2D. Nevertheless, these findings should be interpreted as associations rather than causal effects, and further validation in independent prospective cohorts is needed.

These findings align with a large body of work showing that circulating amino acid patterns, particularly branched-chain and aromatic amino acids, can track insulin resistance and predict future T2D, beyond traditional risk factors (6, 10). Our results extend this concept by focusing on acetylated amino acid species, which may better reflect the balance between amino acid availability, acetyl-CoA flux, and protein turnover. Notably, the opposite directions observed for N-acetyltryptophan versus most other NAcAAs suggest that distinct upstream pathways contribute to the NAcAA pool, and that a single metabolite cannot capture the full metabolic perturbation in T2D.

Our NAcAA findings should be interpreted in the context of prior work. NAcAAs may reflect amino-acid pool changes, acetylation-deacetylation processes, and renal handling. In population metabolomics, several N-acetylated amino acids track closely with kidney function, consistent with substantial renal contributions to their clearance and metabolism (24, 25), and genetic/omics studies further link renal tubular acetylation pathways (including NAT8-related biology) to N-acetylated amino-acid patterns and kidney outcomes (14). Against this background, the distinct behavior of N-acetyltryptophan in our data is consistent with prior evidence linking tryptophan-related metabolism to diabetes risk and metabolic deterioration. Prospective cohort studies have connected microbiota-related metabolites and amino-acid pathways to incident T2D (9, 19). Clinical studies also support disruption of tryptophan catabolism in T2D and related inflammatory states (26, 27). Notably, directionality may differ by biospecimen and disease stage; for example, reduced urinary N-acetyltryptophan has been reported in nascent metabolic syndrome, which could reflect differences in systemic production versus renal excretion (15). For acetylated BCAA and aromatic-AA species, our results complement the well-established association of circulating BCAAs and aromatic amino acids with insulin resistance and future T2D risk (28, 29). The different behavior of acetylated forms may reflect their sensitivity to downstream handling, including renal tubular secretion. Classic renal physiology data also support active elimination of N-acetylphenylalanine (30). Glutamine-related signals are also biologically plausible. Circulating glutamine has shown inverse associations with T2D risk in meta-analyses and has been linked to incretin and insulin responses in human studies (6, 31, 32). N-acetylglutamate is mechanistically connected to mitochondrial nitrogen handling through urea-cycle regulation (33). In addition, urea-cycle suppression has been described in metabolic liver disease, which is closely linked to insulin resistance (34). For N-acetylproline, evidence is more limited in established T2D but it has emerged as a discriminating metabolite in other dysglycaemia-related settings such as gestational diabetes, and proline catabolism is known to influence mitochondrial redox and stress responses (35–37). Finally, modified metabolites such as N6-acetyllysine have been associated with kidney function decline and progression in diabetes-related cohorts (38). This supports the interpretation that acetylated amino-acid signals may capture an early metabolic–renal interface relevant to diabetes phenotypes.

Taken together, our quantitative NAcAA signature supports the view that NAcAAs capture a metabolic–renal interface in T2D. These metabolites may reflect systemic metabolic stress, including amino-acid and tryptophan-related biology. They may also be shaped by renal handling, which could help explain their associations with T2D and their contribution to model performance. Several limitations should nevertheless be considered. First, this cross-sectional design cannot establish temporality or causality. Second, the cohort was drawn from a single setting, and external validation in independent populations is still required to confirm generalizability. Third, NAcAA concentrations may be influenced by kidney function, diabetes duration, antidiabetic medications, and dietary intake. We added descriptive information on diabetes duration and antidiabetic medication use among T2D participants in Table 1. These variables were not incorporated into the primary case–control models because they were disease-specific characteristics applicable only to T2D participants. In addition, detailed dietary intake was not systematically collected, although overnight fasting blood collection was used to reduce acute postprandial effects. Therefore, residual confounding related to medication exposure, disease duration, and habitual diet cannot be excluded. Finally, we quantified NAcAAs in plasma only; paired urine measurements, together with longitudinal and prospective sampling, would be valuable to disentangle altered production from altered clearance and to evaluate whether the NAcAA panel can predict incident T2D and related complications.

In the present study, the NAcAA-based metabolite panel achieved an AUC of 0.963, whereas the combined conventional glycaemic-marker model based on fasting plasma glucose and HbA1c yielded an AUC of 0.908. This comparison provides important clinical context for interpreting the performance of the metabolite-based model. Fasting plasma glucose and HbA1c are well-established clinical indicators for diabetes diagnosis and glycaemic assessment, and their strong discriminative performance was therefore expected. However, these indicators mainly reflect glycaemic status and may be influenced by glucose-lowering therapy, disease duration, recent dietary control, and individual differences in glycaemic management. In contrast, the NAcAA-based metabolite panel may capture broader metabolic perturbations associated with T2D, including amino acid metabolism, acetylated metabolite alterations, and systemic metabolic changes beyond hyperglycaemia. Therefore, the clinical value of the metabolite panel should not be interpreted as replacing fasting plasma glucose or HbA1c, but rather as providing complementary metabolic information. The higher AUC observed for the metabolite panel in this cohort suggests that these metabolites may reflect disease-associated metabolic abnormalities that are not fully represented by conventional glycaemic markers. If validated in independent cohorts, targeted NAcAA profiling may serve as an add-on approach for metabolic risk characterization in research or specialized clinical laboratory settings, although standardization, cost-effectiveness, and clinically actionable thresholds require further investigation.

Future studies should validate the NAcAA signature in multi-centre cohorts and in prospective designs, ideally with repeated measurements to test stability over time and to evaluate prediction of incident T2D and diabetic complications. Mechanistic studies are also warranted to clarify why NAcAAs show heterogeneous associations with glycaemic and renal indices. Genetic and functional evidence suggests that acetylation and deacetylation pathways (e.g., NAT8 and aminoacylase 1) influence circulating and urinary N-acetylated amino acids and relate to kidney outcomes (14). In parallel, renal tubular transporters, including organic anion transporters, participate in the handling of endogenous metabolites—including amino acid derivatives and uremic solutes—which may contribute to the clinical correlations observed in our cohort (39, 40). Finally, combining NAcAA profiling with other layers of data (e.g., gut microbiome, genetics, and proteomics) may help to map the upstream sources of key metabolites such as N-acetyltryptophan and to identify actionable pathways.

## Data Availability

The original contributions presented in the study are included in the article/Supplementary material, further inquiries can be directed to the corresponding authors.

## Supplementary material

SUPPLEMENTARY FIGURE S1 Representative extracted-ion chromatograms of the isotope-labelled internal standards. **(A)** Representative extracted-ion chromatograms of the internal-standard panel acquired in MRM mode. The chromatograms show the retention-time distribution of the stable-isotope-labelled internal standards used for correction of the 19 target NAcAAs during LC–MS/MS quantification.

SUPPLEMENTARY FIGURE S2 Diagnostic performance of the conventional glycaemic-marker model for T2D discrimination. **(A)** Receiver operating characteristic curve for the logistic regression model combining fasting plasma glucose and HbA1c. The model achieved an AUC of 0.908.
