# Supplementary figures and images for: A plasma N-acetyl amino acid panel for type 2 diabetes discrimination built by targeted LC–MS/MS: A case–control study
Source: Front Med (Lausanne). 2026 Jun 3;13:1830709. doi: 10.3389/fmed.2026.1830709 (PMC13272076; doi:10.3389/fmed.2026.1830709)

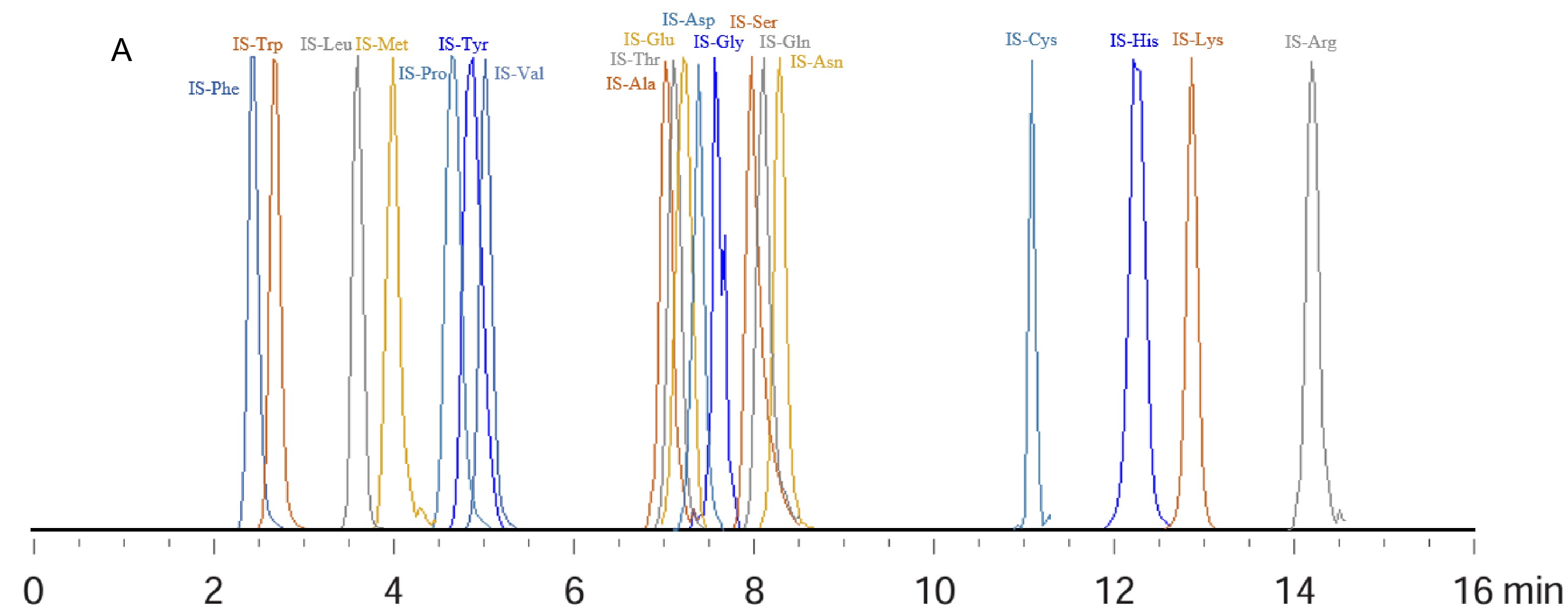

Supplement: SUPPLEMENTARY FIGURE S1 — Representative extracted-ion chromatograms of the isotope-labelled internal standards. (A) Representative extracted-ion chromatograms of the internal-standard panel acquired in MRM mode. The chromatograms show the retention-time distribution of the stable-isotope-labelled internal standards used for correction of the 19 target NAcAAs during LC–MS/MS quantification. [file Image_1.JPEG]

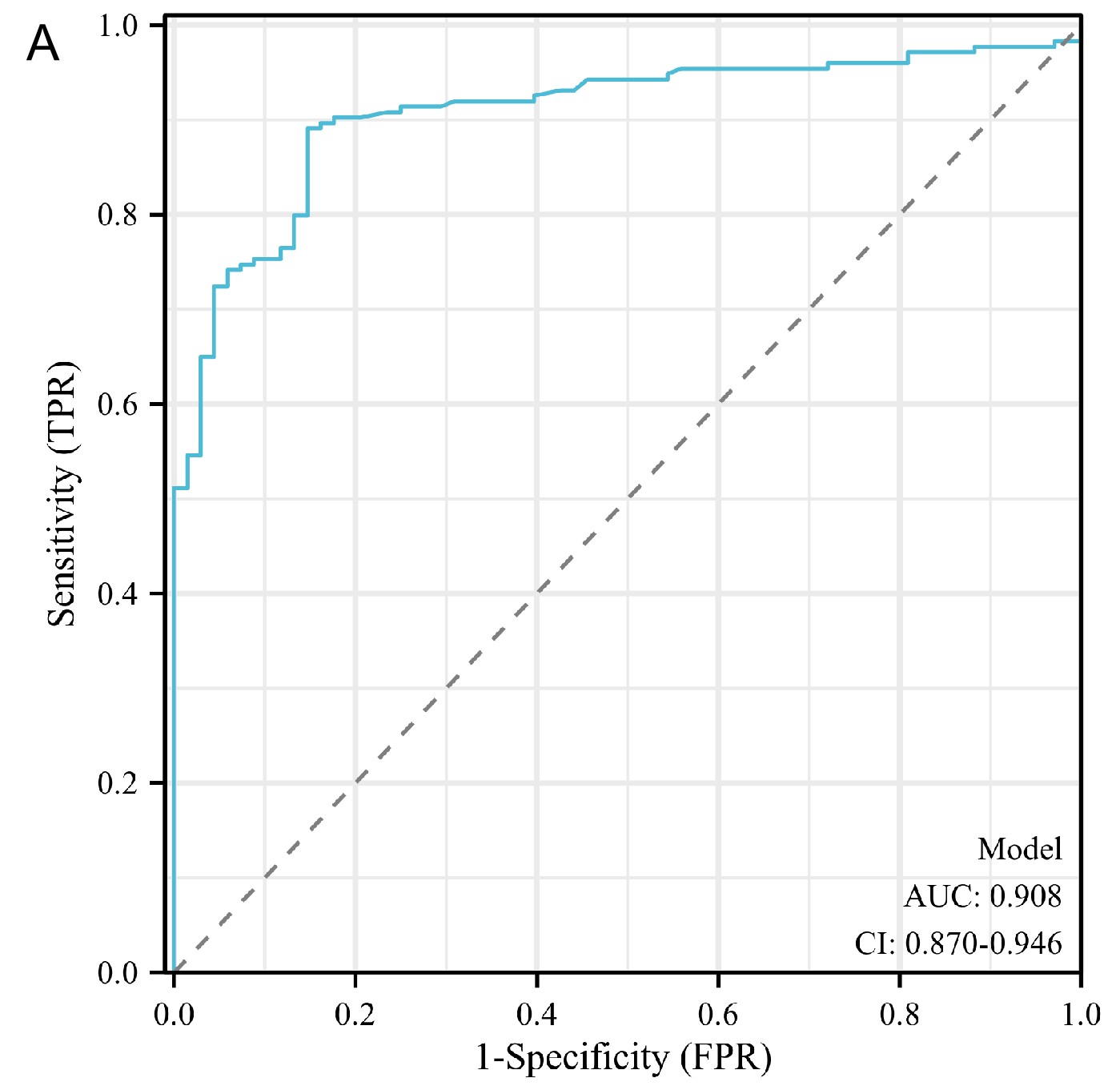

Supplement: SUPPLEMENTARY FIGURE S2 — Diagnostic performance of the conventional glycaemic-marker model for T2D discrimination. (A) Receiver operating characteristic curve for the logistic regression model combining fasting plasma glucose and HbA1c. The model achieved an AUC of 0.908. [file Image_2.JPEG]
